# An Improved Relative GNSS Tracking Method Utilizing Single Frequency Receivers

**DOI:** 10.3390/s20154073

**Published:** 2020-07-22

**Authors:** Wenhao Yang, Yue Liu, Fanming Liu

**Affiliations:** College of Automation, Harbin Engineering University, Harbin 150001, China; irene_16317yang@hrbeu.edu.cn (W.Y.); liuyuehrb@outlook.com (Y.L.)

**Keywords:** ambiguity-free, differential GNSS relative positioning, dead reckoning, relative motion solution, single-frequency receiver

## Abstract

The Global Navigation Satellite Systems (GNSS) becomes the primary choice for device localization in outdoor situations. At the same time, many applications do not require precise absolute Earth coordinates, but instead, inferring the geometric configuration information of the constituent nodes in the system by relative positioning. The Real-Time Kinematic (RTK) technique shows its efficiency and accuracy in calculating the relative position. However, when the cycle slips occur, the RTK method may take a long time to obtain a fixed ambiguity value, and the positioning result will be a “float” solution with a low meter accuracy. The novel method presented in this paper is based on the Relative GNSS Tracking Algorithm (Regtrack). It calculates the changes in the relative baseline between two receivers without an ambiguity estimation. The dead reckoning method is used to give out the relative baseline solution while a parallel running Extended Kalman Filter (EKF) method reinitiates the relative baseline when too many validation failures happen. We conducted both static and kinematic tests to assess the performance of the new methodology. The experimental results show that the proposed strategy can give accurate millimeter-scale solutions of relative motion vectors in adjacent two epochs. The relative baseline solution can be sub-decimeter level with or without the base station is holding static. In the meantime, when the initial tracking point and base station coordinates are precisely obtained, the tracking result error can be only 40 cm away from the ground truth after a 25 min drive test in an urban environment. The efficiency test shows that the proposed method can be a real-time method, the time that calculates one epoch of measurement data is no more than 80 ms and is less than 10 ms for best results. The novel method can be used as a more robust and accurate ambiguity free tracking approach for outdoor applications.

## 1. Introduction

In recent years, novel opportunities are offered for low cost and highly accurate positioning owing to the ability to share Global Navigation Satellite System (GNSS) measurement data between receivers, coupled with the unique constraints of the GNSS positioning domain [1,2,3]. In outdoor applications, such as autonomous driving, collision avoidance, land surveying, precision agriculture, and formation flying of unmanned aerial vehicles (UAVs), the need to obtain a precise relative positioning instead of an absolute positioning result by using GNSS is more prevalent [4,5].

In using the GNSS system for relative positioning, carrier phase is the most accurate measurement method [6,7]. However, it is necessary to determine the ambiguity of the carrier phase measurement, that is, to solve the ambiguity problem [8]. The least-squares ambiguity decorrelation adjustment (lambda) is the best solution widely used at present thanks to its computational efficiency and supports the decorrelation between estimated ambiguities [9,10]. Many applications and studies have used this method to achieve precise positioning. Takasu, T. presented the RTKLIB, which is an open source software that uses LAMBDA algorithm to implement a real-time kinematic (RTK) strategy [11]. Travis et al. proposed a new vehicle path tracking method based on Extended Kalman Filter and LAMBDA method [12]. The LAMBDA method shows its efficiency and accuracy in giving out the precise GNSS position solution. It is often applied on an epoch-by-epoch basis, thus instantaneously, for which the method becomes completely immune for cycle slips [13,14,15]. However, there are still some points needed to be taken care of before using LAMBDA in the RTK method. First, the LAMBDA method needs information about the ambiguity float solution. The appropriate accuracy position is usually needed in the RTK method to avoid the false ambiguity fixing problem before the Extended Kalman Filter bias states have had time to converge. Second, the ambiguity fix-and-hold strategy is usually used for moving rovers in the RTK method. When cycle slips occur, RTK strategy needs to ignore the estimation of satellites with cycle slips, and it takes a long time to obtain and maintain a fixed Ambiguity Resolution (AR) value using LAMBDA algorithm [16]. During this time, on the off chance that the number of satellites with no cycle slips being less than four, the position result will be “float” solution down to meter-level accuracy. There are many cycle slip processing methods in high precision positioning, but before using the “repaired satellites”, there should be an ARlockcnt parameter that specifies how many samples delay occur before a new satellite (or a satellite that just recovered from a cycle-slip) is used for ambiguity resolution. Holding off the use of the new phase-biased estimate from the Kalman filter until it has had enough time to converge prevents the corruption of the ambiguity resolution integer set, which, in turn, prevents a loss of fix or the false fix. In the case of frequent cycle-slips, this could mean loss of fix from having too few satellites available, or it could mean a false fix since fewer satellites give a less robust ambiguity resolution.

The other category is to obtain the relative position solution without solving the ambiguity fixing problem. The ambiguity free strategy shows its advantages over the ambiguity fixing strategy, such as less time required to obtain the exact position solution, and anti cycle slip. The typical algorithm is Ambiguity Function Method [17,18,19,20]. However, this is controversial because there may be several maximum points, and the AFM algorithm must filter the incorrect peak points in the search area to find the best location. Nevertheless, the low computation of the AFM class methods is another block for achieving a real-time positioning [21]. A novel class of integer equivariant methods was introduced by P.J.G. Teunissen [22]. It follows the principle of integer removal recovery and is larger than the class of integer estimation and more meaningful than the class of linear unbiased estimation. This method has also been successfully applied in several studies [23]. Cellmer, S. proposed another method of precise GNSS positioning, named the Modified Ambiguity Fucntion Approach (MAFA). The new processing method is based on the least square adjustment algorithm and does not need the integer search phase. However, the realization of precise positioning needs a high accuracy approximate position [24,25,26,27]. Yang, W. et al. then presented a novel search method based on the Segmented Simulated Annealing and the MAFA method. The computation time is significantly reduced, and the multi-peaks problem is solved using the Kernel Density Estimation method. However, the author claims that a kinematic experiment cannot be achieved owing to the requirement of the accurate relative motion vector between two adjacent epochs [28]. Their following research proposed a precise GNSS tracking method based on the SSA—MAFA method and the relative motion solution method. The novel method can achieve a centimeter-level of accuracy absolute positioning even if the prior position is several meters away from the correct value. However, the total calculation time is still affected by the precision of the initial *z* coordinate [29]. Another idea is to construct a new observation model and directly remove the ambiguity term in the calculation equation. The represented algorithm is the real-time relative tracking method (Regtrack), which uses a new double difference model to eliminate ambiguity term in measurement data and provides a high-precision solution for tracking the relative baseline between two receiving nodes [30]. However, if the number of useful satellites is less than four, this method cannot give a correct solution. Thus the long time tracking result could be poor. To solve the problem, Hedgecock, W. et al. used the Ambiguity Function Method (AFM) to reinitiate the tracking relative baseline vector when too many times of validation failures happen. However, the computation efficiency is too slow, and the number of epochs data spent to give out the correct solution is very significant [31]. The other related work can be found in Reference [32,33,34]. The combined difference square (CDS) observation is proposed to give out an accuracy localization result. The novel method is based on the new observation by eliminating the nonlinear terms as well as the integer ambiguities in the difference square observations. However, multiple epochs of measurement data are required to give out the current solution. Above all the ambiguity-free positioning methods, the Regtrack method can be the most efficient one. It eliminates the ambiguity term by using constant integer nature of ambiguities during two adjacent epochs. Thus no more ambiguities fixing problem should be resolved, and there is no possibility of wrong ambiguity fixation. However, when there are too many cycle slips of synchronization for all satellites, Regtrack method cannot give accurate relative motion solution, and the tracking effect is poor.

In the present paper, a Modified Relative GNSS Tracking (M-Regtrack) method is proposed. The purpose of this method is to obtain a consistent relative positioning accuracy without solving the ambiguity determination problem even when there are too many cycle slips in the measured data. The main contributions of this paper are:

(1) A double difference model spanning receiver and time is proposed to calculate the accurate relative motion vector between two adjacent data periods. The relative baseline tracking solution is given by dead reckoning algorithm. Therefore, an accurate tracking solution can be obtained without solving the ambiguity fixing problem.

(2) While there is a failure of the validation for the relative motion vector, we innovatively take the previous solution as the current one considering the fast sample rate of both receivers, e.g., 1 Hz. Thus, the tracking can be accurate and continuous even if the receiver loses all the satellite signals.

(3) A parallel running Extended Kalman Filter is used to reinitialize the relative baseline vector if too many validation failures occur, which in turn, prevent more accumulation error into the tracking result when using the dead reckoning method, which is often the case by using the Regtrack method. The results show that the tracking solution can be more accurate and robust than without using the EKF method.

The rest of the paper is organized as follows: The proposed algorithm is described in Section 2. The experiment and analysis are introduced in Section 3. The paper concludes in Section 4.

## 2. Methodology

### 2.1. Single Differential Model

The Global Navigation Satellite System receiver provides two types of measurements, pseudo range (code) and carrier phase. The nominal accuracy of carrier phase measurement is about 0.01 cycles, and carrier phase measurement is more robust to multipath error [35,36]. Thus it becomes the relevant observations for precise GNSS positioning. Differential GNSS (DGNSS) method is usually used to eliminate the error sources in the observations, yielding a more precise measurement.

Considering the pseudorange and carrier phase measurements from the same satellite *i* for two GNSS receivers *r* and *b*, the equations of single differentials across receivers can be shown as:

(1) $$\Delta P_{rb}^{i}=\Delta \rho_{rb}^{i}+C\Delta t_{rb}+\epsilon$$

(2) $$\Delta \phi_{rb}^{i}=\Delta \rho_{rb}^{i}+C\Delta t_{rb}+\lambda \Delta N_{rb}^{i}+\epsilon$$

where Δ, single difference operation; *P*, pseudo distance measurement (meters); geometric distance between ρ receiver and satellite (meters); *C*, the speed of light; *t*, receiver clock offset; ϵ, multipath and other unmodeled error sources (meters); ϕ, measured value of carrier phase (meters); *N*, integer ambiguity (in cycle).

**Figure 1:**
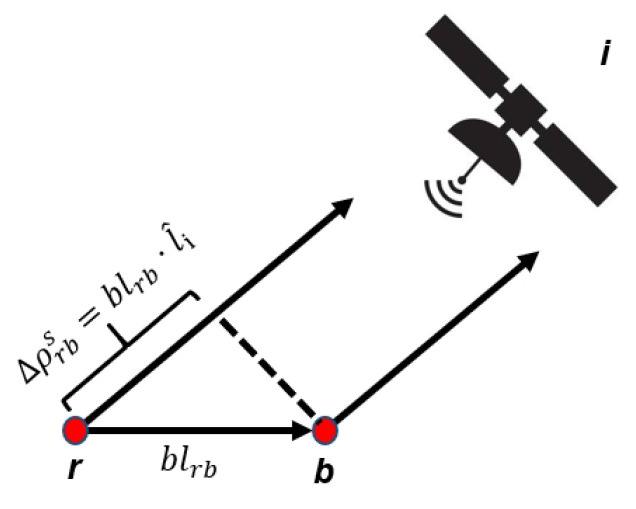
Graphical representation of the relationship between $\Delta \rho_{rb}^{i}$ and $bl_{rb}$ [29].

As shown in the Equations (1) and (2), which eliminate the source of error associated with atmospheric delay and satellite clock offset—the latter is polluted by integer ambiguity. Considering that the satellite is far away from both receivers, it can be assumed that the unit direction vector (from the receiver to the satellite) is the same [30], as shown in Figure 1. Therefore, $\Delta \rho_{rb}^{i}$ can be replaced by the following:

(3) $$\Delta \rho_{rb}^{i}=bl_{rb}\cdot {\hat{l}}_{i}$$

Among them, $bl_{rb}$, the true relative position between the receiver *r* and *b*, is called the baseline vector; ${\hat{l}}_{i}$, the satellite line—of—sight unit vector *i*, and then Equation (2) can be overwritten as:

(4) $$\Delta \phi_{rb}^{i}=bl_{rb}\cdot {\hat{l}}_{i}+C\Delta t_{rb}+\lambda \Delta N_{rb}^{i}+\epsilon$$

Although most errors have been eliminated by utilizing single differentials, there is still a need for double differentials to reduce the remaining errors, e.g., receiver clock bias errors.

### 2.2. Double Differential Model

With multiple satellites in range, double differentials between receivers and satellites ($DD_{R}^{S}$) can be carried out. Take receivers *r*, *b* and satellites *i*, *k* as an example, the double differentials across receivers and satellites can be formed as:

(5) $$\nabla \Delta \phi_{rb}^{ik}=\nabla \Delta \rho_{rb}^{ik}+\lambda \nabla \Delta N_{rb}^{ik}+\epsilon$$

where ∇Δ, double-differencing operation.

Although the $DD_{R}^{S}$ method can eliminate the error of receiver clock offset, the integer ambiguity pollution still exists. Fortunately, if there is no cycle slip, the cycle ambiguity can be a constant between two adjacent observation periods. Cycle slip is a phenomenon that loses satellite locking and cannot return the correct carrier phase observations. Double differential ($DD_{t_{1,2}}$) can be formed between two adjacent epochs $t_{1}$ and $t_{2}$:

(6) $$\begin{array}{cc} \nabla \Delta \phi_{rb}^{i} & =\Delta \phi_{rb}^{i}\left(t_{2}\right)-\Delta \phi_{rb}^{i}\left(t_{1}\right)\\ \; & =\nabla \Delta \rho_{rb}^{i}+C\nabla \Delta t_{rb}+\epsilon \end{array}$$

The new double differential equation ($DD_{t_{1,2}}$) no longer includes an ambiguity item, whereas the receiver clock bias errors only left a drift item $\nabla \Delta t_{rb}$. Moreover, the remaining unmodeled noise is in the granularity of a few milimeter [37]. After ignoring the remaining noise item, Equation (6) can be rewritten based on Equation (3):

(7) $$\nabla \Delta \phi_{rb}^{i}=bl_{rb}\left(t_{2}\right)\cdot {\hat{l}}_{i}\left(t_{2}\right)-bl_{rb}\left(t_{1}\right)\cdot {\hat{l}}_{i}\left(t_{1}\right)+C\nabla \Delta t_{rb}$$

It is proved in [30] that the error introduced in the position domain is 10.636×$10^{-9}$ times the baseline length by assuming the unit direction vectors to a single satellite are identical for receivers throughout a one-time epoch, which is a tiny number when the baseline length is no more significant than 1000 km. Thus, Equation (7) can be updated by:

(8) $$\nabla \Delta \phi_{rb}^{i}=\Delta bl_{rb}\left(t_{1},t_{2}\right)\cdot {\hat{l}}_{i}+C\nabla \Delta t_{rb}$$

It is obviouse that $DD^{t_{1,2}}$ captures a relative motion $\Delta bl_{rb}$ between receiver *r* and *b* within two adjacent epochs.

### 2.3. Relative GNSS Tracking Algorithm Overview

As the $DD^{t_{1,2}}$ model can give out the relative motion between receivers through time, a relative GNSS tracking algorithm (Regtrack) can be obtained [30]. For simplicity, the operator symbol ∇Δ represents the double difference between receivers and time, as shown in the following equations. Suppose there are *n* satellites in sight (elevation angle ≥$15^{\circ }$), then the observation system equations can be obtained by using Equation (6):

(9) $$\Phi =\rho +C\times t_{rb}$$

with

(10) $$\Phi =\left[\begin{array}{c} \nabla \Delta \phi_{rb}^{1}\\ \nabla \Delta \phi_{rb}^{2}\\ \vdots \\ \nabla \Delta \phi_{rb}^{n} \end{array}\right],\rho =\left[\begin{array}{c} \nabla \Delta \rho_{rb}^{1}\\ \nabla \Delta \rho_{rb}^{2}\\ \vdots \\ \nabla \Delta \rho_{rb}^{n} \end{array}\right]$$

A Taylor series expansion of Equation (9) can be represented as:

(11) e=δ−Bb

with

(12) $$\delta =\left[\begin{array}{c} \nabla \Delta \phi_{rb}^{1}-\nabla \Delta \rho_{rb}^{1}\left(x_{t_{0}}\right)+C\nabla \Delta t_{e_{0}}\\ \nabla \Delta \phi_{rb}^{2}-\nabla \Delta \rho_{rb}^{2}\left(x_{t_{0}}\right)+C\nabla \Delta t_{e_{0}}\\ \vdots \\ \nabla \Delta \phi_{rb}^{n}-\nabla \Delta \rho_{rb}^{n}\left(x_{t_{0}}\right)+C\nabla \Delta t_{e_{0}} \end{array}\right]$$

(13) $$B=\left[\begin{array}{cccc} \frac{\partial \nabla \Delta \rho_{rb}^{1}}{\partial x} & \frac{\partial \nabla \Delta \rho_{rb}^{1}}{\partial y} & \frac{\partial \nabla \Delta \rho_{rb}^{1}}{\partial z} & 1\\ \frac{\partial \nabla \Delta \rho_{rb}^{2}}{\partial x} & \frac{\partial \nabla \Delta \rho_{rb}^{2}}{\partial y} & \frac{\partial \nabla \Delta \rho_{rb}^{2}}{\partial z} & 1\\ \vdots & \vdots & \vdots & \vdots \\ \frac{\partial \nabla \Delta \rho_{rb}^{n}}{\partial x} & \frac{\partial \nabla \Delta \rho_{rb}^{n}}{\partial y} & \frac{\partial \nabla \Delta \rho_{rb}^{n}}{\partial z} & 1 \end{array}\right]$$

where *e*, error vector (n×1); *b*, increment vector based on prior coordinates $x_{t_{0}}$; *B*, design matrix (n×4); δ, misclosure vector (n×1); $x_{t_{0}}$, a prior position of rover receiver; $t_{e_{0}}$, the initial clock bias error of receivers.

Then the adjustment problem can be formed as:

(14) $$\arg\min_{b}(e^{T}We)$$

where *W* represents the weight matrix, which can be formed as:

(15) $$W=P^{-1}$$

with

(16) $$P=R_{t_{1}}+R_{t_{2}}$$

(17) $$R=\left[\begin{array}{cccc} \sigma_{1}^{2}\\ \; & \sigma_{2}^{2}\\ \; & \; & \ddots \\ \; & \; & \; & \sigma_{n}^{2} \end{array}\right]$$

where $\sigma_{n}$ is the standard deviation of phase measurement errors of *n* satellites. The calculation formula is as follows:

(18) $$\sigma_{n}^{2}=2\left(a^{2}+b^{2}/sin^{2}El^{n}+c^{2}\right)+d^{2}$$

where a,b,c, carrier phase error coefficient, in meters; El, satellite elevation angle; *d*, satellite clock error, in meters.

The solution to this problem is to use the following parameter vectors:

(19) $$b=-\lambda {\left(B^{T}WB\right)}^{-1}B^{T}W\delta$$

Since *b* represents the relative motion of two receivers between two adjacent time periods, a simple dead reckoning method (adding the relative motion solution to the last relative position estimate) can be used to obtain the relative position of each epoch between two receivers. It should be noticed that the position of the referenced receiver is not required to be a high degree of accuracy; even the reference receiver is not stationary, as the solution, *b* represents the change of the baseline vector between two receivers in two adjacent epochs. In the meantime, the error introduced in the relative solution *b* is approximately given by:

(20) $$|\delta {\vec{r}}_{j}|\times |\Delta {\vec{r}}_{i,j}|\times 10^{-8}$$

where $\delta {\vec{r}}_{j}$, the error of the prior position of the reference receiver referred to the correct coordinates; $\Delta {\vec{r}}_{i,j}$, the relative position vector between the rover receiver and the referenced receiver. As a result, if the position error of the referenced receiver was 100 m, for example, the error of the relative position solution could be no larger than one centimeter even if the baseline vector between two receivers is 10 km apart.

### 2.4. The Modified Relative Tracking Approach

**Figure 2:**
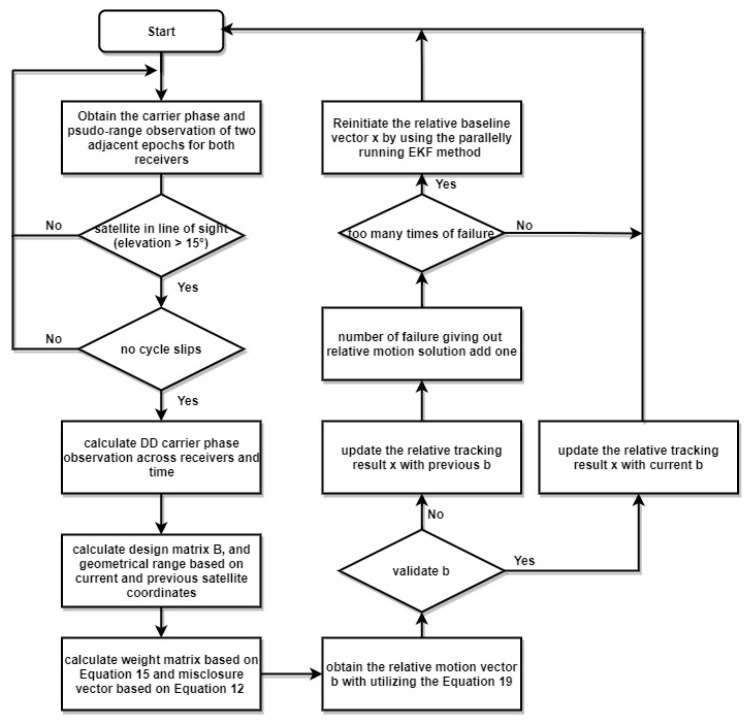
The whole procedure of the M-Regtrack algorithm.

The Regtrack algorithm can achieve a very accuracy tracking result. However, it suffered from one serious limitation, if the number of useful satellites was down below four at any point during the tracking procedure, without a reinitialization, the long time tracking result could be poor [31]. Thus we presented the modified relative tracking method (M-Regtrack), the whole algorithm of the M-Regtrack procedure is shown in Figure 2.

After getting the solution vector *b*, the verification process is performed. The program checks whether the number of useful satellites is more significant than 4 and confirms that the solution is under the settled threshold value.

Recall the hypothesis we make in Section 2.2. If there is no cycle slip in the carrier phase measurement, the whole cycle ambiguity should be a constant. However, the losses of satellite locks may occur owing to the complex environment. Thus, there is a chance for the validation failure of solution *b*, a simple way to give out the relative motion in this situation is using the previous relative motion vector, considering the sample rate is fast enough. Meanwhile, a parallel running EKF method based on carrier phase measurement and pseudorange measurement can be used to reinitiate the relative baseline vector if the too many times of solution validation failure. The equations of the EKF method are as follows with the very first relative baseline vector $x_{t_{0}}$ that can be obtained by using the Single Point Positioning (SPP) method.

(21) $${\overset{˘}{x}}_{t_{i}}=F_{t_{i-1}}^{t_{i}}{\hat{x}}_{t_{i-1}}$$

(22) $${\overset{˘}{P}}_{t_{i}}=F_{t_{i-1}}^{t_{i}}{\hat{P}}_{t_{i-1}}{F_{t_{i-1}}^{t_{i}}}^{T}+Q_{t_{i-1}}^{t_{i}}$$

(23) $${\hat{x}}_{t_{i}}={\overset{˘}{x}}_{t_{i}}+K_{t_{i}}\left(y_{t_{i}}-h\left({\overset{˘}{x}}_{t_{i}}\right)\right)$$

(24) $$K_{t_{i}}={\overset{˘}{P}}_{t_{i}}H_{t_{i}}\left({\overset{˘}{x}}_{t_{i}}\right){\left\{H_{t_{i}}\left({\overset{˘}{x}}_{t_{i}}\right){\overset{˘}{P}}_{t_{i}}H_{t_{i}}{\left({\overset{˘}{x}}_{t_{i}}\right)}^{T}+R_{t_{i}}\right\}}^{-1}$$

(25) $${\hat{P}}_{t_{i}}=\left(I-K_{t_{i}}H_{t_{i}}\left({\overset{˘}{x}}_{t_{i}}\right)\right){\overset{˘}{P}}_{t_{i}}$$

where symbol $\overset{˘}{\left(\cdot \right)}$ and $\hat{\left(\cdot \right)}$, before and after measurement update of EKF; $x_{t_{i}}$, the unknown state vector (relative baseline vector and rover velocity) at time $t_{i}$; $P_{t_{i}}$, covariance matrix for $x_{t_{i}}$; $K_{t_{i}}$, the Kalman gain matrix at time $t_{i}$; $y_{t_{i}}$ and h(x), $DD_{R}^{S}$ measurement vector and measurement model vector at time $t_{i}$; $R_{t_{i}}$, covariance matrix of measurement errors, which is shown as Equation (17); H(x), the matrix of partial derivatives; $F_{t_{i-1}}^{t_{i}}$ and $Q_{t_{i-1}}^{t_{i}}$, the transition matrix and the covariance matrix of the system noise from epoch time $t_{i-1}$ to $t_{i}$. The equations for $F_{t_{i-1}}^{t_{i}}$ and $Q_{t_{i-1}}^{t_{i}}$ are as follows,

(26) $$F_{t_{i-1}}^{t_{i}}=\left[\begin{array}{cc} I_{3\times 3} & I_{3\times 3}\tau_{r}\\ \; & I_{3\times 3} \end{array}\right]$$

(27) $$Q_{t_{i-1}}^{t_{i}}=\left[\begin{array}{cc} 0_{3\times 3}\\ \; & Q_{v} \end{array}\right]$$

where:

(28) $$Q_{v}=E_{r}^{T}\left[\begin{array}{ccc} \sigma_{v_{e}}^{2}\tau_{r}\\ \; & \sigma_{v_{n}}^{2}\tau_{r}\\ \; & \; & \sigma_{v_{u}}^{2}\tau_{r} \end{array}\right]E_{r}$$

where $\tau_{r}$, GNSS receiver sampling interval (in seconds); $\sigma_{v_{e}},\sigma_{v_{n}},\sigma_{v_{u}}$, the standard deviations of east, north and up components of the rover velocity noise.

## 3. Experiments and Analysis

The validity of this method is verified by static and kinematic experiments. In the static experiment, the accuracy of the relative motion solution was tested. Next, we conducted three kinematic experiments to prove the tracking accuracy of the M-Regtrack method. The first kinematic experiment was used to compare the tracking accuracy between Regtrack and M-Regtrack when both receivers were moving. The second experiment was used to test the trajectory tracking accuracy when one receiver was holding static without knowing its precise coordinate. In this test, we compared the trajectory tracking result between Regtrack and M-Regtrack. The last experiment was used to prove the tracking accuracy when the coordinates of the referenced station were known prior, and the initial relative baseline vector between receivers was calibrated in advance. The tracking result of Regtrack and M-Regtrack were compared, referenced to the ground truth. For all the kinematic experiments, we used the RTK result as the reference value (the LAMBDA method gives out the ambiguity value). We set the parameter filter type of the RTK method to the combination mode of “forward” and “backward”, and get the reference value. It should be mentioned here that we did not use the new GLONASS FDMA model for giving out the reference value. However, the new model can be used to give integer ambiguity solutions with high success rates, and now and in the future it offers an excellent opportunity for the new integer estimable GLONASS model to utilize combined FDMA−CDMA signals [38,39].

### 3.1. Static Experiment

**Figure 3:**
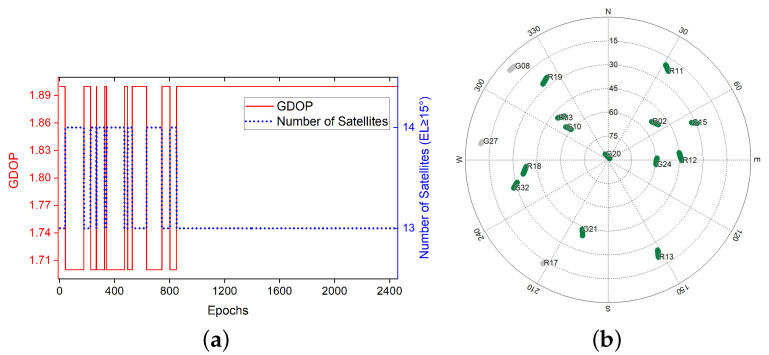
Geometric Dilution Precision (GDOP) and Sky Map during data sampling of experiment Section 3.1 (**a**) GDOP values and number of satellites. (**b**) Sky map of visible satellites, with a gray track indicating that the satellite’s altitude is less than $15^{\circ }$ [29].

We carried out the static test in a parking lot at Queen’s University in Kingston, Ontario, utilizing two u-Blox Neo-M8T and low-cost single frequency receiver antennas. The receiver was connected to the notebook via a universal serial bus with a sampling rate set to 5 Hz. The experiment was carried out at 00:06:26 (Global Positioning System Time) on 18 August 2019. Data of L1 frequency GPS and GLONASS signals were collected over 2449 periods.The GDOP and sky map during the sampling period is displayed in Figure 3.

**Figure 4:**
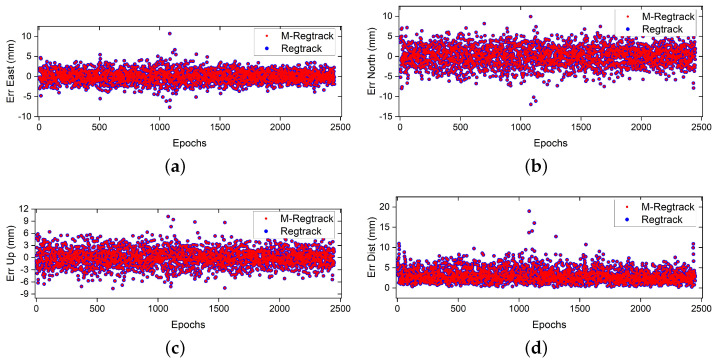
Error result of relative motion solution (**a**) East component error. (**b**) North component error. (**c**) Error in up component. (**d**) Error of relative motion solution, where Dist = $\sqrt{err_{E}^{2}+err_{N}^{2}+err_{U}^{2}}$ [29].

As shown in Figure 3b, there were no cycle slips that happened during the sampling periods. Since the two receivers remained stationary, the relative motion should be zero. Therefore, any value except zero in the solution should be regarded as an error. The error results during the sampling periods are shown in Figure 4.

Since there is no cycle slip between two adjacent data periods in this test, the relative motion vector solutions have all passed the verification process. The results show that both M-Regtrack method and Regtrack method can give the relative motion results of each epoch, and the accuracy is equivalent to millimeter level.

### 3.2. Kinematic Experiment

#### 3.2.1. Kinematic Experiment When Both Receivers Are Moving

**Figure 5:**
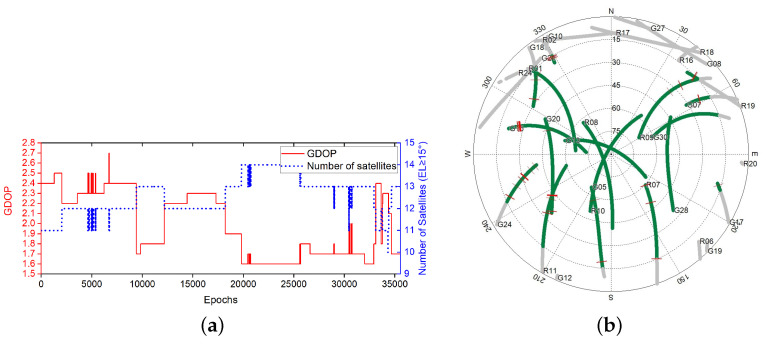
Geometric Dilution Precision (GDOP) and Sky Map during data sampling of experiment Section 3.2.1 (**a**) GDOP values and number of satellites. (**b**) Sky map of visible satellites, with a gray track indicating that the satellite’s altitude is less than $15^{\circ }$, and the red line indicating cycle slips.

In this experiment, two Emlid Reach NEO-M8T receivers were installed at both ends of the canoe (at 2.82 m). The canoe sailed in the ocean near Sussex, England, and the sampling rate of the receiver was set at 5 Hz. The experiment was conducted at 09:17:54, 3 April 2016 (GPS time), and 35,545 periods of data were collected from GPS and GLONASS signals with L1 frequency. The GDOP and sky map during the sampling period is displayed in Figure 5.

In the test, the mobile receiver can be regarded as a static reference station (even if it is not), leading to all relative motion attributable to the second receivers with a fixed distance (2.82 m). The direction between them changes with the movement of the platform. Therefore, the solution should be a circle with a radius equal to the distance between two receivers. Because cycle slips rarely occur during data sampling periods, the number of verification failures of relative motion vector solution was very small, as shown in Table 1.

**Figure 6:**
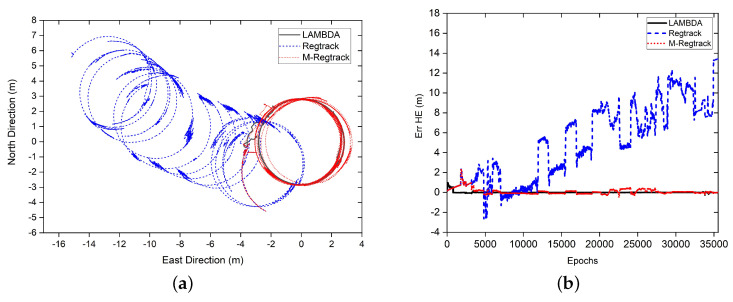
Relative tracking results and corresponding horizontal errors. (**a**) The relative tracking result of one receiver to another (both receivers are moving). (**b**) Horizontal error result, in which the distance between two receivers is 2.82 m, where $HE=\sqrt{err_{N}^{2}+err_{E}^{2}}$.

It is shown in Figure 6a that both the LAMBDA method and the M-Regtrack method can give out a beautiful circle trajectory while the Regtrack method failed to give out a complete circle. The reason for the Regtrack method not giving out the correct solution is owing to the too long time accumulation of the motion vector error as well as the failure of giving out the correct solution for participate epochs of data. In contrast, the M-Regtrack can converge to the correct solution by using the EKF method to initial to the correct one during the too long time of validation failure. It is more evident from the horizontal error result, shown in Figure 6b, that the proposed method can achieve a submeter track result for the whole tracking period while the Regtrack method diverges to almost 14 m away. It should be noticed that the M-Regtrack method uses a dead reckoning method without solving the ambiguity fixing problem.

#### 3.2.2. Kinematic Experiment When One Receiver Is Holding Static without Knowing Its Precise Coordinates

**Figure 7:**
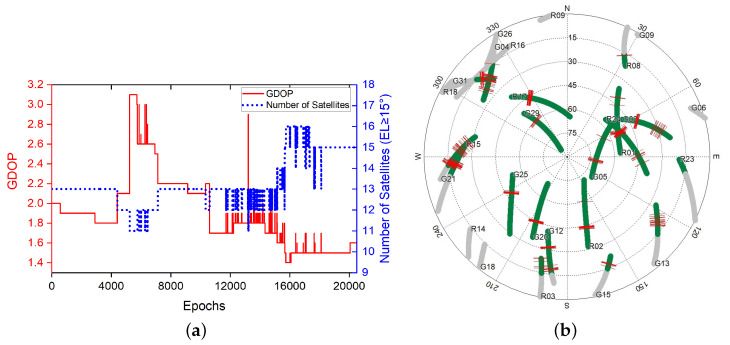
Geometric Dilution Precision (GDOP) and Sky Map during data sampling of experiment Section 3.2.2 (**a**) GDOP values and number of satellites. (**b**) Sky map of visible satellites, with a gray track indicating that the satellite’s altitude is less than $15^{\circ }$, and the red line indicates cycle slips.

In this test, one Emlid Reach NEO-M8T single frequency receiver was mounted on top of a car, and the other Emlid Reach NEO-M8T receiver was holding static on the ground as a base station. As the precise position of the base station was not used, this experiment was used to test the tracking accuracy other than the absolute positioning. The experiment was carried out on 2016/7/11 23:55:15 (GPS Time). A total of 20,493 epochs of data were sampled with a sample rate of 5 Hz for GPS and GLONASS L1 frequency signals. The GDOP and sky map during the sampling period is displayed in Figure 7.

In this experiment, there were basically no trees or high buildings blocking the satellite signal. However, there were a little too many simultaneous cycle slips on all satellites at the beginning and the middle sampling periods of the moving M8T receiver, which made it a little more challenging for giving out a high accuracy tracking result. The validation status of the motion vector solutions is shown in Table 2.

**Figure 8:**
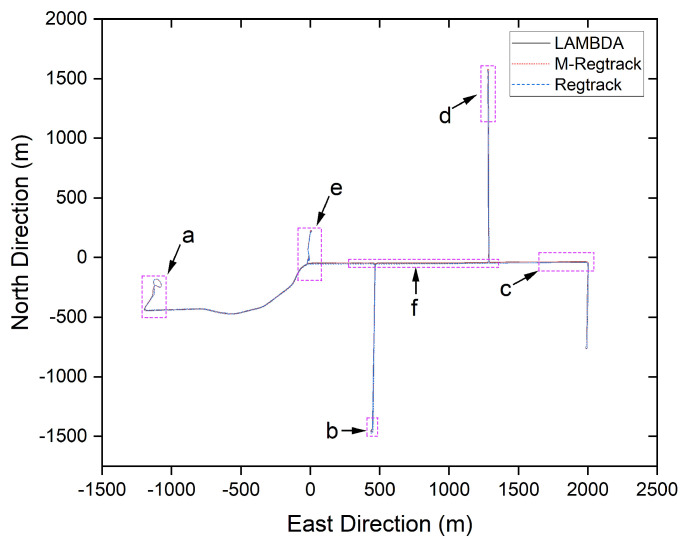
The trajectory tracking result for one rover receiver towards a static receiver without knowing the precise coordinates of the static one, where a–f indicates Figure 9a–f, respectively.

**Figure 9:**
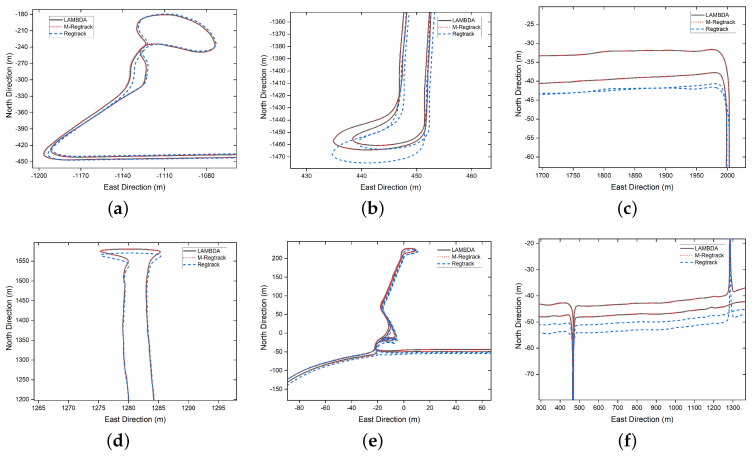
Enlarged part of the trajectory tracking result, where (**a**–**f**) represents the enlarged part of Figure 8a–f.

There was a total 0.6% of the epochs failing to give out the correct relative motion vector solution and the previous solution was used to calculate the relative tracking result. In the M-Regtrack method, after detecting too much time of validation failure, it used the EKF method to reinitiate the start point of the corresponding tracking result for the continuous calculations; the tracking result of the experiment is shown in Figure 8 and Figure 9.

**Figure 10:**
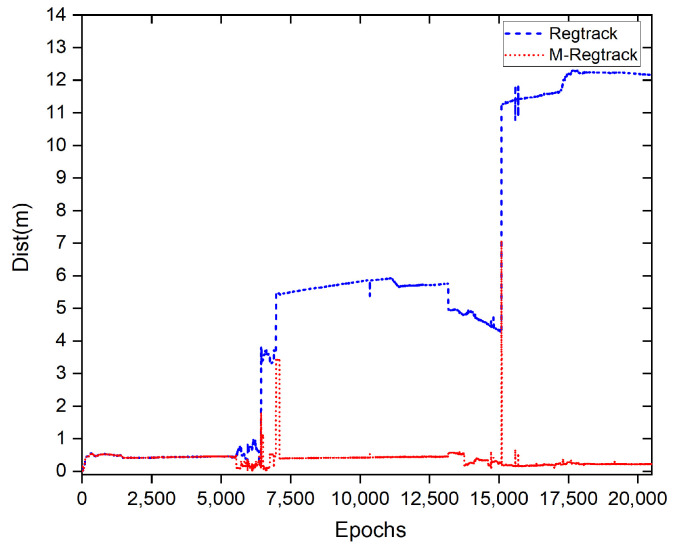
The trajectory tracking error result of different methods referenced to the solution of the LAMBDA method, where Dist = $\sqrt{err_{E}^{2}+err_{N}^{2}+err_{U}^{2}}$.

The M-Regtrack method can be much more accurate than the Regtrack method. The main reason is the use of the EKF method in the M-Regtrack method for reinitialization after a too long time validation failure. As shown in Figure 10, the M-Regtrack can give out a submeter or even subdecimeter tracking result for most of the experiment periods while the Regtrack method diverged to 12 m away from the genuinely relative trajectory, even though both methods used a dead reckoning method to calculate the tracking result.

#### 3.2.3. Kinematic Experiment When One Receiver Is Holding Static Knowing Its Precise Coordinates

**Figure 11:**
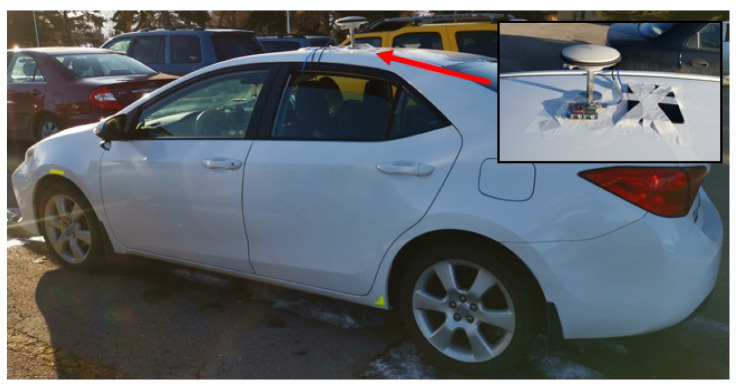
The experimental platform, where one uBlox-F9 receiver is installed on the top of the experimental vehicle.

**Figure 12:**
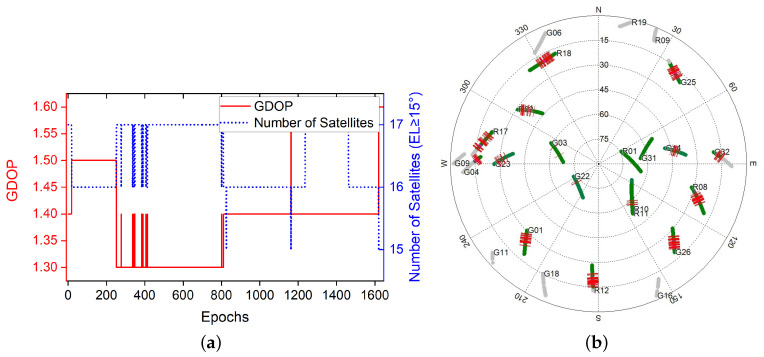
Geometric Dilution Precision (GDOP) and Sky Map during data sampling of experiment Section 3.2.3 (**a**) GDOP values and number of satellites. (**b**) Sky map of visible satellites, with a gray track indicating that the satellite’s altitude is less than $15^{\circ }$, and the red lines indicate cycle slips.

In this test, we used a uBlox-F9 receiver as the rover receiver and installed it on the top of the car, as shown in Figure 11. The Trimble-R9 receiver is used as a base station placed in a fixed position, where the receiver can obtain high-quality satellite signals. The exact position of the base station in the ECEF frame was (−1,641,890.0811, −3,664,879.3446, 4,939,969.4285). The experiment was conducted at Calgary University, Alberta, Canada at 21:20:50 (GPS time) on 25 November 2019. For GPS and GLONASS L1 frequency signals, 1637 periods of data are sampled at a sampling rate of 1 Hz. The sky map and GDOP during the sampling periods are displayed in Figure 12.

The precise position of the start point for the rover receiver was calibrated manually at the beginning. Thus, the solution was compared with the absolute ground truth. As shown in Figure 12, there were too many simultaneous cycle slips on all satellites in this experiment. The main reason is that the high buildings or the tree beside the roads blocked the satellite signal, which made it very challenging to give out a high accuracy absolute tracking result.

**Figure 13:**
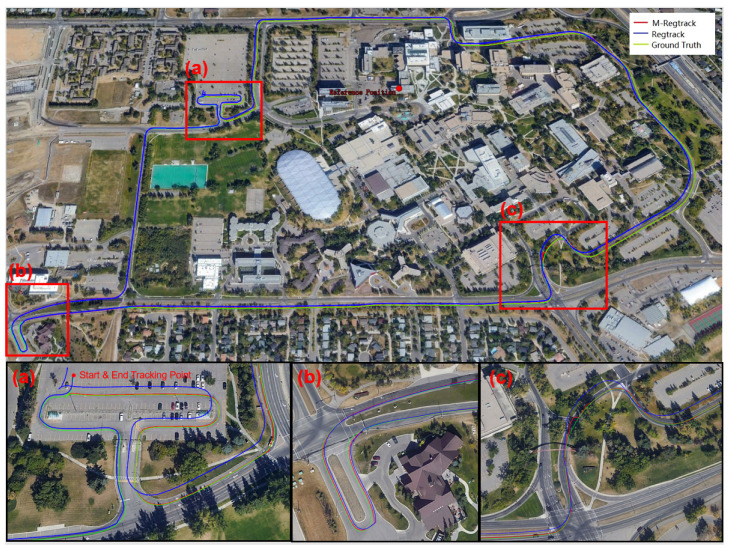
The ground truth map and the tracking result comparison of different methods for the moving car experiment Section 3.2.3, where the red dot was the reference receiver, the (**a**–**c**) indicate the enlarged parts.

**Figure 14:**
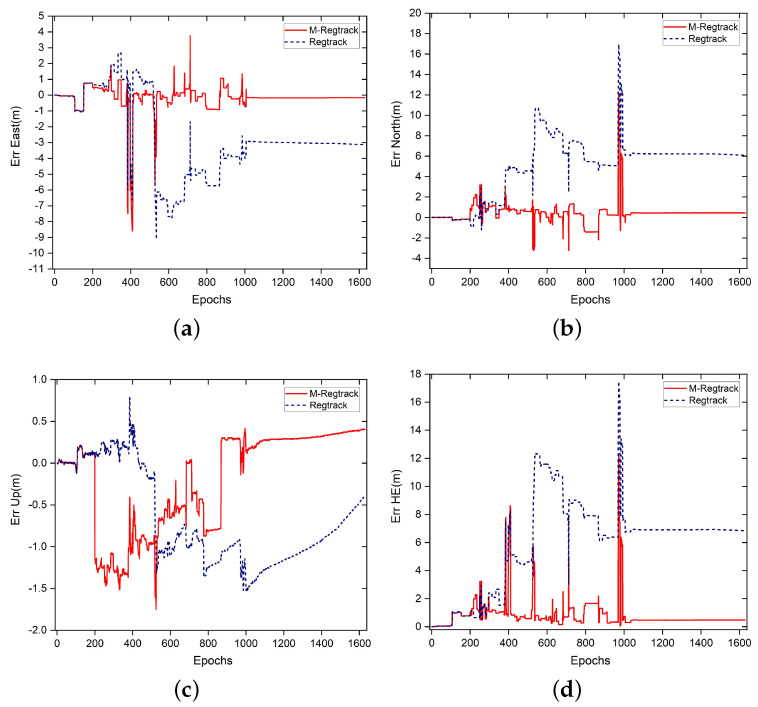
Error of absolute tracking result. (**a**) East component error. (**b**) North component error. (**c**) Up component Error. (**d**) Horizontal error results, where $HE=\sqrt{err_{N}^{2}+err_{E}^{2}}$.

It is shown in Figure 13 that the M-Regtrack method can be more robust than the Regtrack method and converge to the correct solution by utilizing the EKF method when too many failures of obtaining the correct relative motion solution and the failure rate reached 8.5%, as shown in Table 3. The tracking error result is shown in Figure 14, the M-Regtrack can give out the tracking solution at the endpoint with no greater than 48 cm of horizontal error after a 25 min drive test. In contrast, the Regtrack method diverged to about 6 m away from the ground truth. The accuracy for most of the absolute tracking results of M-Regtrack can be submeter level. However, there were still some epochs in which the tracking result was several meters away from the ground truth owing to the too many cycle slips shown in Figure 12. The M-Regtrack can be a very robust approach and it can be a Lane-level tracking method for outdoor applications as long as the precise coordinates of the initial point can be obtained both for the rover and the base station receiver.

### 3.3. Efficiency of the M-Regtrack Method

To analyze the scalability and processing requirements of the proposed method, we compared the calculation efficiency for both methods in the dynamic test. The test used a desktop computer equipped with Intel Core i7-6700T CPU, 16GB memory and running Windows 10 operating system.

**Figure 15:**
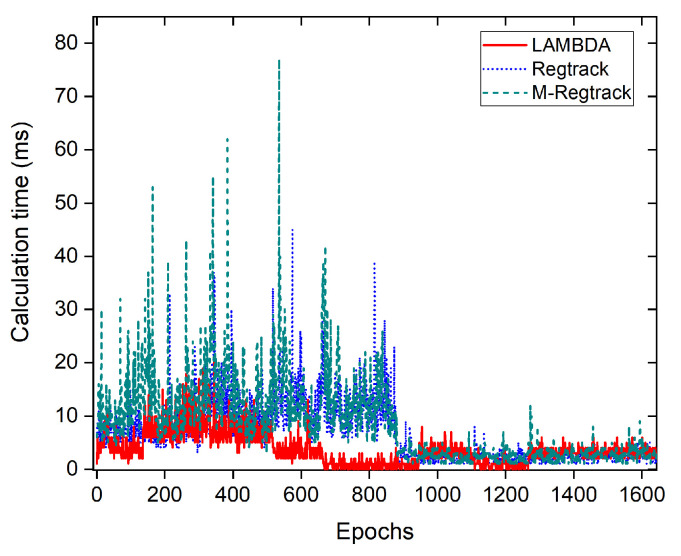
Calculation time in the kinematic experiment.

As is shown in Figure 15, both methods can achieve a real-time solution. The LAMBDA method can give out the relative baseline solution very efficiently when the ambiguities are fixed. The M-Regtrack method can be less efficient than the Regtrack method owing to the use of the EKF method to do the reinitialization of the relative baseline vector. The computation time of the novel method can also be closely related to the number of satellites in line of sight without cycle slips. Although our new method is slightly lower than the LAMBDA method efficiency, the calculation time depends on many factors (i.e., the degree of optimization code of the program), which has nothing to do with the nature of each method.

## 4. Conclusions

In order to establish a more robust, accurate, and inexpensive relative positioning platform, we propose a method to track the relative baseline variations between two receivers without solving the ambiguity fixing problem. The method we propose is called the Modified Relative GNSS Tracking Method (M-Regtrack).

In the proposed method, the common noise sources are reduced or eliminated by using the single differential method. A double differential model over receivers and time is created by differencing the same single difference observable in the next epoch. The constant nature of the ambiguity term *N* is used in the time double differential model to remove it altogether. In addition, the projection relative to the change of baseline vector can be geometrically expressed as a direction vector from the receiver to the satellite. Using the least square method and high quality carrier phase, the relative motion vector of each epoch can be accurately estimated without solving the ambiguity fixing problem. If the number of useful satellites is less than four, and the validation of the relative motion solution fails, the previous correct solution is used as the current one considering the sample rate is fast enough. If there are too many validation failures, the parallelly running EKF method based on the carrier phase measurements and the result of Single Point Positioning (SPP) is used to reinitiate the relative baseline vector to converge to the correct tracking result. Static experiments show that, like Regtrack method, the proposed method can give the relative motion solution in millimeter scale. In the three kinematic experiments, the M-Regtrack method shows its robustness against the Regtrack method. When two receivers are both moving, the tracking result of the M-Regtrack method can be a perfect circle where the radius of the circle is the distance between two receivers while the Regtrack method fails to give out a complete circle. When one receiver is holding static, the tracking result error of the M-Regtrack method could be submeter or subcentimeter accuracy during all the phases of kinematic experiments, which is a vast improvement over the Regtrack method. Nevertheless, when the precise coordinate of the base station is obtained, and the prior position of the rover receiver is calibrated, the tracking result error of the M-Regtrack method can be only 40 cm after a 25 min driving test compared with the ground truth while the Regtrack method diverged to 6 m away. The efficiency experiment also shows the M-Regtrack can be an efficient real-time tracking method.

The M-Regtrack method shows its accuracy and efficiency even after a long time drive experiment in an urban environment. However, it is still an open problem for the M-Regtrack to obtain the precise initial point efficiently at start-up time instead of manually calibrating for both the relative and absolute tracking solution. Recent advances in research on obtaining the precise initial point are based on the MAFA method, but only the static experiment is carried out. We plan to continue to study this problem and use M-Regtrack and MAFA to realize a complete independent positioning system.

## Figures and Tables

**Table 1:** The validation status of the relative motion vector solutions: kinematic experiment Section 3.2.1.

	Total Number of Relative Motion Vector Solution	Number of Solution Validation Failure	Number of Reinitializing the Relative Baseline by Using EKF
M-Regtrack	35,545	11	2
Regtrack	35,545	11	0

**Table 2:** The validation status of the relative motion vector solutions: kinematic experiment Section 3.2.2.

	Total Number of Relative Motion Vector Solution	Number of Solution Validation Failure	Number of Reinitializing the Relative Baseline by Using EKF
M-Regtrack	20,493	136	27
Regtrack	20,493	136	0

**Table 3:** The validation status of the relative motion vector solutions: kinematic experiment Section 3.2.3.

	Total Number of Relative Motion Vector Solution	Number of Solution Validation Failure	Number of Reinitializing the Relative Baseline by Using EKF
M-Regtrack	1637	140	28
Regtrack	1637	140	0

## Abbreviations

The following abbreviations are used in this manuscript:

AR	Ambiguity Resolution
AFM	Ambiguity Function Method
CDS	Combined Difference Square
DD	Double Differential
GDOP	Geometric Dilution of Precision
GNSS	Global Navigation Satellite System
LAMBDA	Least-squares Ambiguity Decorrelation Adjustment
MAFA	Modified Ambiguity Function Approach
M-Regtrack	Modified Relative GNSS Tracking
RTK	Real-Time Kinematic
SPP	Single Point Positioning
